# Comprehensive Assessment of Eutrophication in Xiamen Bay and Its Implications for Management Strategy in Southeast China

**DOI:** 10.3390/ijerph192013055

**Published:** 2022-10-11

**Authors:** Yang Luo, Jin-Wen Liu, Jian-Wei Wu, Zheng Yuan, Ji-Wei Zhang, Chao Gao, Zhi-Yu Lin

**Affiliations:** 1 College of Environmental Science and Engineering, Ocean University of China, Qingdao 266100, China; 2 Third Institute of Oceanography, Ministry of Natural Resources, Xiamen 361005, China; 3 Xiamen Environmental Monitor Station, Xiamen 361021, China

**Keywords:** coastal eutrophication, “phase II” methods, ASSETS, area ratio method, Xiamen Bay

## Abstract

The eutrophication of coastal water has been a critical environmental problem in China’s offshore areas. How to effectively assess the status of coastal waters is key for pollution treatment and environmental protection. In recent years, eutrophication-symptom-based and multi-indicator methods, termed “phase II” methods, have been gradually adopted to assess the eutrophication status in some coastal waters in China and have achieved success. The cumulative quantile is typically selected to determine the characteristic value of an indicator in “phase II” methods. The influence of small-scale damaged water bodies on eutrophication assessment may be exaggerated, which often leads to the overassessment of the eutrophication status. In this study, the area ratio method was integrated into the assessment of the estuarine trophic status (ASSETS) method in order to assess the eutrophication status of Xiamen Bay in 2016. The results indicated that, in 2016, the eutrophication status of Xiamen Bay coastal waters was moderate and exhibited spatiotemporal variation. The area ratio method can effectively reduce the effect of small-scale coastal waters with extremely high eutrophication on the overassessment of eutrophication at the broader scale, allowing the eutrophication status to be better reflected, even with limited observation data. The centralized distribution of pollution sources and poor hydrodynamic conditions are the main reasons for the aforementioned phenomenon. Controlling the pollution discharge from the Jiulong River in flood seasons is key to reducing eutrophication in Xiamen coastal waters.

## 1. Introduction

Eutrophication is a phenomenon in which the accelerated growth of algae in seawater caused by nutrient enrichment in water interferes with the balance and stability of the marine ecosystem, affecting water quality [1]. The main cause of eutrophication is an abnormal increase in the primary productivity of waters [2]. In recent decades, the problem of eutrophication in offshore areas has been one of the severe marine environmental problems that beset coastal countries around the world [3]. Human activities have intensified the transport of nutrient elements such as nitrogen and phosphorus to oceans, causing changes in the composition and degradation of functions of the ecosystem, and a series of problems such as ecological risk disasters that severely threaten the health of the ecological environment of coastal waters [4,5,6,7,8,9].

At present, the phase I assessment method of nutrient-based eutrophication has been widely used in the study of the eutrophication assessment of coastal waters in China [10,11,12,13,14,15,16]. This assessment method constructs a linear empirical model of nitrogen, phosphorus, chemical oxygen demand (COD), and chlorophyll-a concentration to deal with the response of all ocean systems [17]. The most representative methods include the EI, NQI, TRIX, and space methods [2,18,19]. The above assessment systems tend to overemphasize the influence of nutrients in characterizing the eutrophication status, simplify the response correlation between nutrient concentration and phytoplankton biomass, and ignore the differences in the sensitivity and response of different sea areas to nutrients [20,21]. Therefore, the results obtained consistently show that the near-shore areas with higher nutrient concentrations are areas with a severe eutrophication status. Such assessment systems often obtained unexplainable results indicating severe eutrophication symptoms under low-nutrient concentration conditions or the absence of any eutrophication symptoms under high-nutrient concentration conditions [22,23,24]. Therefore, domestic academic circles have been paying attention to eutrophication-symptom-based and multi-indicator methods, termed “phase II” methods [25], such as the assessment of the estuarine trophic status [17,26,27], the Water Framework Directive [28] and the Oslo–Paris Convention for the Protection of the North Sea Comprehensive Procedure [29].

In recent years, Chinese researchers improved “phase II” methods in various ways and performed application demonstrations in Bohai Bay [30,31], Yangtze River Estuary [32,33], Beibu Gulf [11], Jiaozhou Bay [21], Maowei Sea [34], Daya Bay [35], Xiamen Bay [36], and Taiwan [37]. Most domestic studies took the arithmetic mean value of monitoring the results from all stations in the study area or the concentration value corresponding to a certain cumulative percentage as the eutrophication indicator eigenvalue. This method is better suited for those bays with high water exchange capacity, insignificant spatiotemporal variation in water quality damage, and numerous monitoring stations. However, for those bays with complex pollution mechanisms and significant regional differences in water quality damage, it tends to exaggerate the influence of the affected local areas in the process of eutrophication assessment. In these cases, the eigenvalue method is not as scientific as the area ratio method in eutrophication assessment. In this study, we (1) integrate the area ratio method into the assessment of the estuarine trophic status (ASSETS) method to (2) assess the coastal eutrophication of Xiamen sea areas, (3) analyze the main causes, and (4) propose the key approach for nutrients discharge control. These attempts are made to improve the ASSETS method and provide a more reasonable eutrophication status of Xiamen Bay.

## 2. Study Area and Data Collections

### 2.1. Study Area

Xiamen Bay is located in the subtropical areas of the southeast coast of China. According to the currently adjusted functional zoning of the marine environment in Xiamen, Xiamen Bay covers a total area of approximately 355 km^2^, which is divided into two parts according to location and geographical characteristics, namely, the inner and outer bays [38]. The inner bays include Tong’an Bay, Maluan Bay, the western sea area, and Jiulong River Estuary; the outer bays include the southern sea area, the eastern sea area, and the Dadeng sea area [39]. The total length of the coastline is approximately 262 km, and as long as 43 km of this length is the deep-water shoreline with a water depth deeper than 10 m. The tidal pattern belongs to the regular semi-diurnal tide, with an average tidal range of approximately 3.96 m. The tide, a reciprocating current, flows into the bay at high tide and out of the bay at low tide [40].

### 2.2. Data Collection

**Figure 1:**
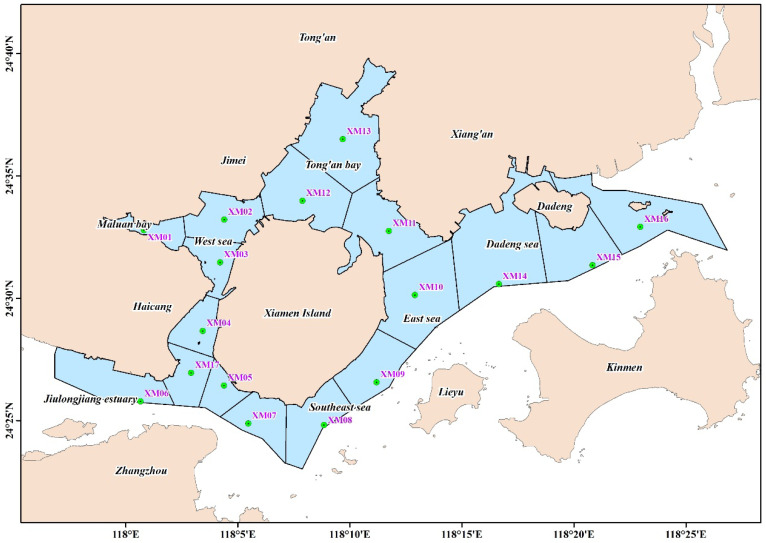
Study area and sampling sites.

We collected the annual water quality monitoring data from a total of 17 monitoring stations in Xiamen Bay in 2016 from the Xiamen Environmental Monitoring Station, including 8 stations, namely, XM03, XM06, XM07, XM08, XM11, XM12, XM14, and XM16, that monitored the area once a month and only collected samples from the surface water. The remaining nine stations monitored the area once a quarter (January, May, August, and October) and collected samples from the surface and bottom water at the same time (only the surface water samples were collected for the XM02 station owing to the shallow water depth). During the quarterly monitoring, the surface and bottom water samples were also collected for analysis at monthly monitoring stations. The layout of the stations is shown in Figure 1, and the coordinates of the stations are shown in Table 1. The indicators selected in this study include chemical oxygen consumption, dissolved oxygen (DO) at the bottom, inorganic phosphorus, total inorganic nitrogen, and chlorophyll-a. Each indicator was monitored and analyzed in accordance with the Specification for Marine Monitoring GB17378.4-2007 (The State Bureau of Quality and Technical Supervision, 2007a) and the Specifications for Oceanographic Survey GB/T12763.4-2007 (The State Bureau of Quality and Technical Supervision, 2007b). The number of occurrences, area, and duration of red tides was obtained from the *Xiamen Municipal Marine Environmental State Bulletin* in previous years. The source was reliable, covering all sea areas in Xiamen Bay, with complete time series during the year.

## 3. Methodology Description

### 3.1. Selection of Assessment Indicators

The eutrophication assessment indicators were divided into three primary indicators, namely, pressure, state, and response, and several secondary indicators were selected for specific characterization under each primary indicator.

Pressure indicator: The pressure indicator of eutrophication established in this study was used to describe the environmental pressure on waters caused by the increase in nutrients in coastal waters. Considering the fact that the relevant eutrophication parameters in the Seawater Quality Standard [41] of China only include COD, dissolved inorganic nitrogen (DIN), and dissolved inorganic phosphorus (DIP), in this study, we used these three parameters as the secondary indicators for “pressure”.

State indicator: According to the theory of the ASSETS method, the eutrophication status of a sea area is measured by the strength of eutrophication symptoms shown in the target sea area, instead of the amount of nutrient salts, and progressively divided into primary and secondary symptoms based on different categories, reflecting the different stages and severities of the eutrophication process [11,41]. Primary symptoms refer to the accelerated growth of algae caused by eutrophication; secondary symptoms refer to the outbreak of algae growth, degrading and consuming the dissolved oxygen at the bottom after the death and sedimentation of organisms, and threatening the survival of benthic organisms [33,42]. At present, marine monitoring is primarily based on chemical indicators and ecological monitoring in China, with no tracing survey of marine organisms [32]. Therefore, water quality and the ecological indicators related to seawater eutrophication were selected as assessment elements as far as state factors are concerned. In this study, we selected the chlorophyll-a concentration at the surface layer as the primary symptom indicator and the amount of dissolved oxygen at the bottom layer and the red tide state as the secondary symptom indicators.

Response indicator: To achieve real-time management and targeted remediation, it is necessary to predict and assess the changes in the eutrophication of sea areas in the next few years. Therefore, in this study, we selected the annual change rate of nutrient emissions in the future as the response indicator.

### 3.2. Grading and Scoring of Indicators

To reflect the equal importance of the secondary indicators and facilitate the rational grading of the primary indicators, we divided the secondary indicators into five degrees: low, medium–low, medium, medium–high, and high. Each degree was scored as 5, 4, 3, 2, and 1, respectively. See Table 2 for details.

### 3.3. Criteria for Grading Indicators

#### 3.3.1. Pressure Indicator

We referred to the study results by Jiang et al. (2014) [36] for the grading standard of the pressure indicator; that is, the improved formula of nutritional state indicator (P) was used to assess the pressure degree, and the assessment results were divided into five degrees based on the mean value method. The calculation formula of the nutritional state indicator is as follows:

(1) $$P=\frac{COD\times DIN\times DIP}{COD^{\prime }\times DIN^{\prime }\times DIP^{\prime }},$$

where *P* represents the nutritional state’s indicator value, which is dimensionless; COD, DIN, and DIP represent the measured values of the chemical oxygen consumption, inorganic nitrogen, and inorganic phosphorus, respectively, measured in mg/L; COD′, DIN′, and DIP′ represent the threshold values of various indicators in Xiamen Bay, which are 3, 0.3, and 0.03 mg/L, respectively.

#### 3.3.2. State Indicator

Chlorophyll-a: According to the recommendations of the American NEEA/ASSETS, when the chlorophyll-a concentration is lower than 5 μg/L, the water body is regarded as being in the optimal eutrophication status [17,43]. Some Chinese studies show that, when the chlorophyll-a concentration is higher than 20 μg/L, the water body is regarded as being about to break out or having been broken out red tides [44,45,46]. The previous research results of eutrophication assessment in Xiamen Bay indicate that, when the chlorophyll-a concentration is higher than 15 μg/L, red tides are prone to break out in the Tong’an Bay of Xiamen [47,48,49]. Therefore, in this study, 5 and 20 μg/L were, respectively, used as the lower and upper limits for the grading standard of the chlorophyll-a concentration indicator, which was divided into five degrees according to the mean value method.

Dissolved oxygen at the bottom layer: It is widely believed internationally that dissolved oxygen falling below 2 mg/L in the water will cause the death of fish and benthic organisms and that below 5 mg/L will cause the pressure effect [50,51]. Therefore, in this study, 5 and 2 mg/L were, respectively, used as the upper and lower limits for the grading standard of the amount of dissolved oxygen at the bottom layer, which were divided into five degrees according to the mean value method.

Red tide state: The grading standard recommended by NEEA/ASSETS was used, and the frequency and duration of red tides in sea areas were assessed in a 1-year cycle.

#### 3.3.3. Response Indicator

The estimated annual change rate of nutrient emission in five years was considered to be the response indicator according to previous domestic and local studies [36,52].

All of the grading standards and values of various assessment indicators are shown in Table 2.

### 3.4. Division of Assessment Units and Determination of Indicator Characteristic Values

Division of assessment units: Xiamen Bay was divided into 17 assessment units, with each survey station as the center, using the ArcGIS software and the Tyson polygon analysis method (Figure 1). The area and proportion of each assessment unit are shown in Table 1.

Characteristic values of assessment unit indicators: In this study, we used the mean value method to determine the characteristic values of the assessment units and analyze the distribution characteristics of each indicator throughout the year and each season to explore the causes of eutrophication. We collected the data from the *Xiamen Municipal Marine Environmental State Bulletin* over the years and assessed the red tide status and the indicators of future nutrient change rate areas. The characteristic values of the assessment units were not calculated separately.

The characteristic values of the assessed sea area indicators: The area ratio method was used to determine the characteristic values of the assessed sea area indicators. That is, first, the characteristic values of each secondary indicator of each assessment unit were calculated, and then the characteristic values of each secondary indicator in the entire Xiamen Bay were calculated according to the area ratio of each assessment unit. The calculation formula is as follows:

(2) $$L=\sum_{i=1}^{n}\;(\frac{S_{i}}{S}C_{i}),$$

where *L* represents the characteristic value of a secondary indicator in the assessed sea area; *S_i_* represents the representative area of the assessment unit *i*, km^2^; *S* represents the total area of Xiamen Bay, km^2^; *C_i_* represents the characteristic value of a secondary index of the assessment unit *i*; and *n* represents the number of assessment units (17 units in total).

### 3.5. Comprehensive Assessment and Final Grading of Eutrophication

Each secondary indicator was graded according to the grading assessment standard, and the score was calculated. The score of each primary indicator depended on the score of the secondary indicator with the lowest score. Matrix analysis was performed on the final scores of each degree of indicator to determine the final eutrophication status. See Table 3 for details.

## 4. Results and Discussion

### 4.1. Pressure Assessment of Eutrophication

**Figure 2:**
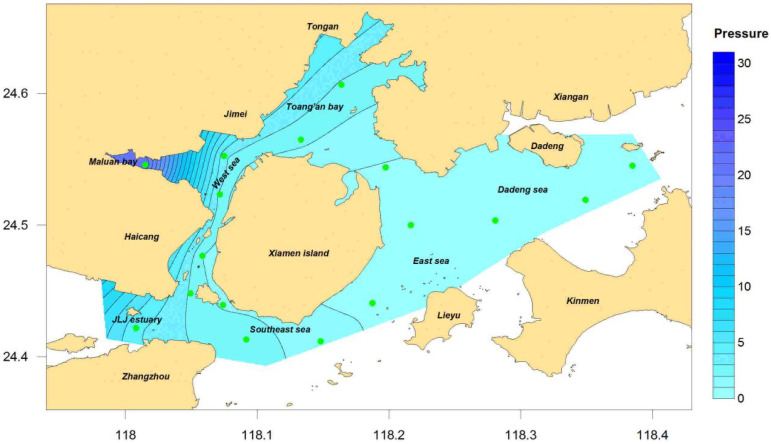
Pressure of eutrophication in Xiamen Bay, 2016.

**Figure 3:**
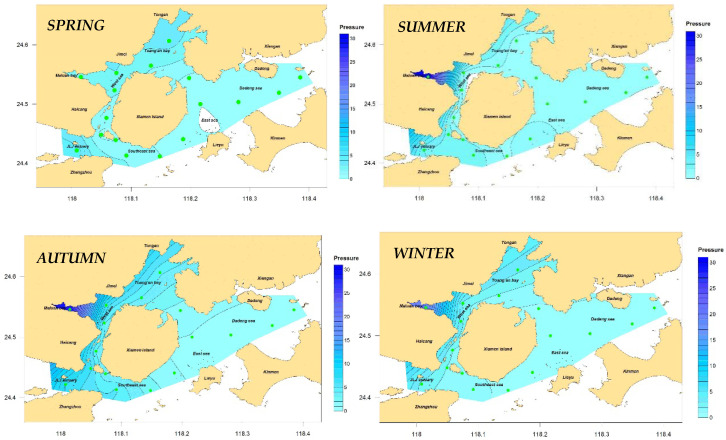
Pressure of eutrophication in Xiamen Bay, seasonal.

The annual distribution results of the eutrophication indicator in Xiamen Bay (Figure 2) and seasonal changes (Figure 3) were calculated on the basis of the annual and quarterly mean values of each station and according to the improved nutritional state indicator Formula (1). In general, the eutrophication status in the Xiamen sea areas in 2016 was moderate, with an indicator value ranging from 0.14 to 0.87 and a mean value of 2.96, showing obvious spatiotemporal variation characteristics.

From the spatial distribution viewpoint, the distributions of the eutrophication indicators in each season were similar and the same as that of the entire year: The indicators in the sea areas of the west and north were higher than those of the east and south. The area with high eutrophication pressure throughout the year and in each season was located in the Maluan Bay and Jiulong River Estuary areas, and that with low pressure was located in the Dadeng sea area. The differences in the eutrophication pressure values of various stations in the entire sea area were the lowest in the spring (all below 4.5), and those of the other three seasons varied significantly. The eutrophication status of Maluan Bay (XM1 station) was the highest among all the stations—several times or even 10 times that of the other stations; this result is not surprising because it has been reported in several studies such as those of Jiang [36], Chen [38], and Chen [47] et al. One of the main reasons for this phenomenon was that the main sources of pollution in Xiamen Bay were concentrated in the western and northern sea areas, which received large amounts of pollution discharge [47]. According to the 2016 statistics of pollution discharge from Xiamen Bay into the open sea provided by the Xiamen Municipal Ocean Development Bureau, the main sources of pollution concentrated in the western and northern sea areas of Xiamen included the Jiulong River, eight streams flowing into the sea, and six sewage treatment plants and other major pollution sources entering the sea. The amount of pollution discharged into the sea accounted for more than 98% of marine pollution in Xiamen waters; more than 85% comes through the mouth of the Jiulong River, which was the main source of pollution discharge in the area. Another reason was that the hydrodynamic conditions of the seas in the west and north were significantly worse than those in the east, with the most manifested in Maluan Bay [39]. The hydrodynamic conditions in this area were the worst among all the analyzed sea areas, with insufficient dissolved oxygen content in the water and its COD concentration exceeding the standard caused by the failure of the degradation of a large number of organic substances. This caused severe eutrophication problems [38].

From the perspective of changes over time, the eutrophication of Xiamen Bay was the most severe in autumn, followed by summer and winter, and the least severe in spring, exhibiting obvious flood season characteristics. According to Figure 3, the maximum value of the eutrophication pressure of the Jiulong River Estuary in the western sea area of the year appeared in summer and autumn, indicating that the concentration and the total number of pollutants discharged in the Jiulong River Basin during the flood seasons were significantly higher than those during the non-flood seasons, so controlling the pollution discharge from the Jiulong River Basin during the flood season is key to reducing the eutrophication of Xiamen Bay.

According to Formula (2), the characteristic value of the eutrophication pressure indicator in Xiamen Bay was 1.99. According to Table 2, the indicator degree was determined to be “high” and scored one point. The top five stations that contributed the most were XM01, XM13, XM06, XM12, and XM02, contributing 66% in total. See Table 4 for details.

### 4.2. State Assessment of Eutrophication

#### 4.2.1. Primary Symptom Assessment

**Figure 4:**
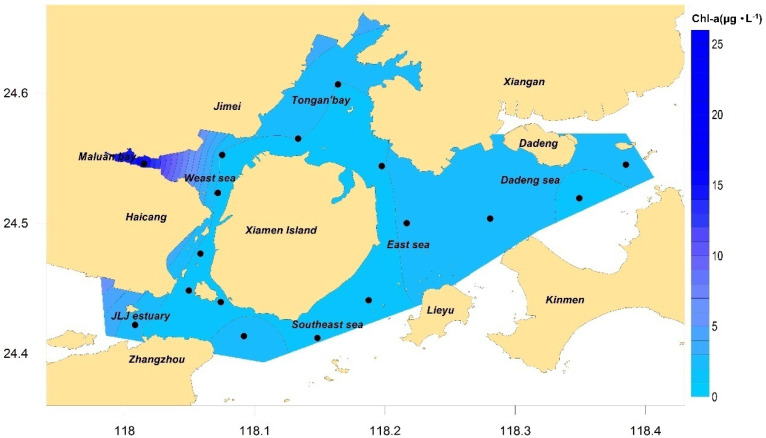
Concentration of chl−a in Xiamen seas, 2016.

**Figure 5:**
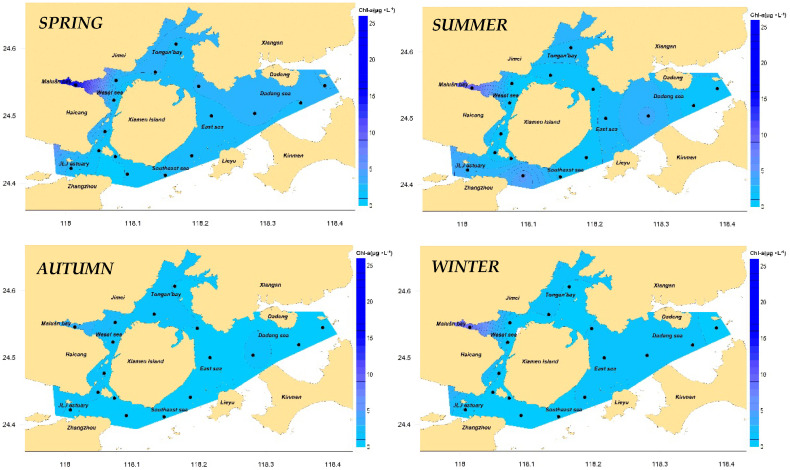
Concentration of chl−a in Xiamen seas, seasonal.

The results of the annual mean and seasonal chlorophyll-a concentration distribution in Xiamen waters are shown in Figure 4 and Figure 5, respectively. The results show that the annual mean chlorophyll-a concentration of the stations in Xiamen Bay was between 1.17 and 15.23 μg/L, with a mean of 2.68 μg/L; chlorophyll-a concentrations in autumn and winter were significantly lower than those in spring and summer; the maximum chlorophyll-a concentration in all seasons appeared in Maluan Bay, with its chlorophyll-a concentration in three seasons (except autumn) significantly higher than that in other areas. The main reason why the chlorophyll-a concentration of Maluan Bay far exceeded that of the other areas was that the bay receives a large number of nutrients discharged from the land [47]. Owing to its shallow water depth, narrow bay mouth, extremely poor exchange capacity of the waters, and long retention time of nutrients, this area provided sufficient nutrients for phytoplankton growth [48]. By analyzing the dissolved oxygen content in the surface water of Maluan Bay, it was found that the dissolved oxygen content in the surface water of this station was the highest among all sea areas in all seasons, and it was significantly positively correlated with the chlorophyll-a concentration. Therefore, it is speculated that strong photosynthesis occurred in the phytoplankton in this area in all seasons, and the released oxygen molecules significantly increased the dissolved oxygen content in the area.

According to Formula (2), the characteristic value of chlorophyll-a concentration in Xiamen Bay was 2.27 μg/L. According to Table 2, the indicator degree was determined to be “low” and scored five points. The top five stations that contributed the most were XM14, XM01, XM13, XM10, and XM16, contributing 55% in total.

#### 4.2.2. Secondary Symptom Assessment

**Figure 6:**
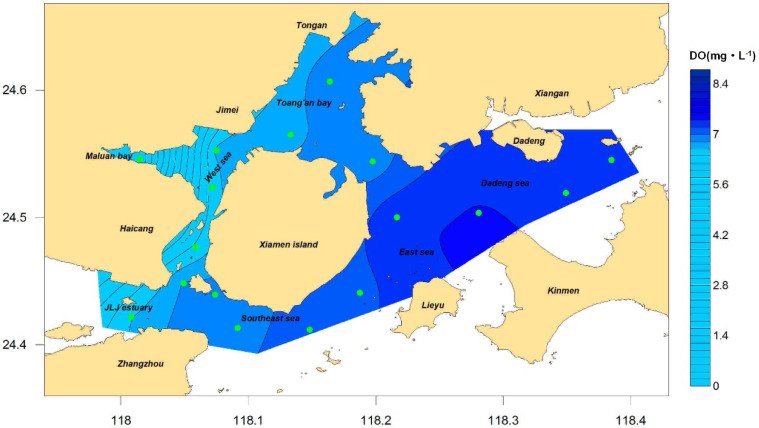
Concentration of bottom DO in Xiamen seas, 2016.

**Figure 7:**
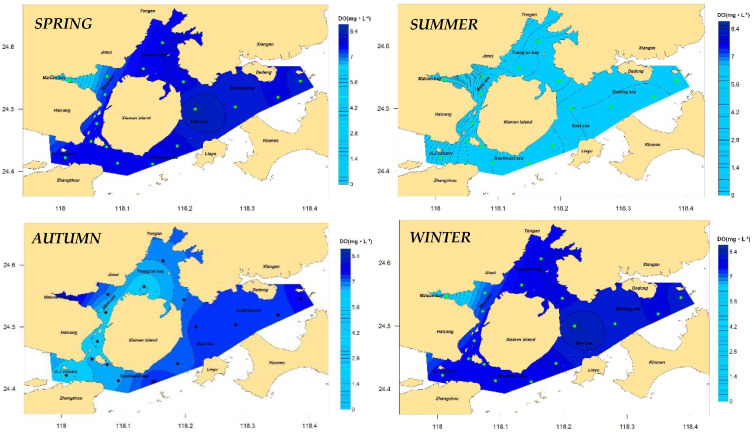
Concentration of bottom DO in Xiamen seas, seasonal.

Dissolved oxygen at the bottom layer: The results of the distribution of the dissolved oxygen concentration at the bottom layer in Xiamen waters throughout the year and each season are shown in Figure 6 and Figure 7, respectively. The results show that the annual dissolved oxygen concentration at the bottom layer in Xiamen Bay was between 4.19 and 7.37 mg/L, with a mean value of 6.77 mg/L, and that of the western sea area was lower than that of the eastern sea area, with the lowest value appearing in Maluan Bay. In general, the increasing order of the concentration values in seasons was winter, autumn, spring, and summer. However, the distribution characteristics of each season were similar, especially in Maluan Bay, where the concentration value in autumn was higher than that in winter, and the minimum value of all sea areas throughout the year (0.46 mg/L) occurred in summer. According to Formula (2), the characteristic value of the dissolved oxygen content at the bottom layer of Xiamen Bay was 6.97 mg/L. According to Table 2, the indicator degree was determined to be “low” and scored five points. The top five stations that contributed the most were XM14, XM13, XM10, XM16, and XM12, with a total contribution of 51%.

Red tides: According to the *2016 Xiamen Municipal Marine Environmental State Bulletin*, there was a red tide and a critical red tide process in the sea areas around Xiamen Island in 2016: From February 18 to March 14, a red tide called *Akashiwosanguinea* occurred in the north Tong’an Bay and Wuyuan Bay; on July 18, a critical red tide reference value of phytoplankton density was recorded in the north Tong’an Bay and Wuyuan Bay, with *Skeletonemacostatum* as the dominant species. In 2016, the accumulation time of red tides in Xiamen Bay was shorter than a month. According to Table 2, the indicator degree was determined to be “medium” and scored three points.

#### 4.2.3. Results of State Assessment

In summary, the scores of the three secondary indicators of chlorophyll-a, dissolved oxygen at the bottom layer, and red tide were five, five, and three, respectively. According to the minimum score principle, the final score of the eutrophication status indicator in Xiamen Bay in 2016 was three points.

### 4.3. Response Assessment of Eutrophication

According to the *Xiamen Municipal Marine Environmental State Bulletin* over the years, the change rate of the nutrient discharge to these sea areas from 2016 to 2021 was analyzed using 2016 as the base year. According to the *2016 Xiamen Municipal Marine Environmental State Bulletin* [53], the total discharge of pollutants into the sea from the main sources of pollution in Xiamen Bay such as the Jiulong River, the major streams of the island, sewage treatment plants, and key rain and sewage mixed outlets in 2016 was 2.93 × 10^5^ tons, with the COD being 1.99 × 10^5^ tons, the total nitrogen 7.82 × 10^4^ tons, and the total phosphorus 3.68 × 10^3^ tons. According to the *2017 Xiamen Municipal Marine Environmental State Bulletin* [54], the total amount of pollution discharged into the open sea from the main pollution sources in Xiamen Bay in 2017 was 1.973 × 10^5^ tons, with the COD being 1.47 × 10^5^ tons, the total nitrogen 4.12 × 10^4^ tons, and the total phosphorus 1.56 × 10^3^ tons, which is a year-on-year decrease by 32.67%, 29.13%, 47.32%, and 57.61%, respectively, from those of 2016. In 2017, Xiamen municipality issued the Implementation Plan for the Pilot Work of Controlling Total Pollutant Discharge from the Jiulong River–Xiamen Bay, which put forward the target requirements for the total pollutant discharge into the open sea, achieving remarkable results. It is foreseeable that in the next five years, the total number of nutrients entering the open sea from Xiamen Bay will continue to decrease, with the overall decrease rate exceeding 10%. According to Table 2, the indicator degree was determined to be “low” and scored five points.

### 4.4. Results of Comprehensive Assessment

According to the above results, the assessment results of the three primary indicators of the eutrophication pressure, state, and response in Xiamen Bay were one, three, and five, respectively. According to Table 3, the final eutrophication status in Xiamen Bay in 2016 was determined to be medium.

### 4.5. Implications for Management Strategies

Based on the above results, it is easy to infer that Maluan Bay and Jiulong River Estuary are the worst areas of eutrophication and, therefore, require stricter measures to improve water quality. For Maluan Bay, the limitation of nutrient input and improvement in the hydrodynamic environment are priority targets; some effective measures include promoting the treatment standards of nearby sewage treatment plants, dredging in an appropriate way, and the remediation of illegal pollution sources. For the Jiulong River Estuary, flood seasons are key to reducing the eutrophication status of Xiamen Bay. Therefore, some agricultural activities are not recommended when close to flood season such as intensive and extensive fertilization in the Jiulong watershed, some measures are advocated for implementation in the long term, such as the establishment of the best management plan and low-impact development.

## 5. Conclusions

(1) The grading results of the three indicators of eutrophication pressure, state, and response in Xiamen Bay in 2016 were high, medium, and low, with score points of one, three, and five, respectively, and the final eutrophication grading result was medium.

(2) The characteristic values of the indicators calculated using the area ratio method were all significantly less than those calculated using the mean value method, indicating that the area ratio method can effectively reduce the impact of the high overall assessment results caused by individual high-value data and improve the scientific nature of the overall assessment results of eutrophication in the case of insufficient observational data.

(3) The eutrophication status in Xiamen Bay was generally high, with obvious spatiotemporal variation characteristics. The eutrophication status in the seas in the west and north was higher than that of the seas in the east and south. The concentrated distribution of pollution sources and poor hydrodynamic conditions were the main reasons for this phenomenon. The eutrophication status was the most severe in autumn and the least severe in spring, exhibiting obvious flood season characteristics. Therefore, controlling the pollutant discharge from the Jiulong River Basin during flood seasons is key to reducing the eutrophication status of Xiamen Bay.

(4) Maluan Bay was the area with the most severe eutrophication in Xiamen Bay. The concentrations of the surface chlorophyll-a and dissolved oxygen in the surface layer were significantly higher than those in other areas in all seasons, whereas the concentration of dissolved oxygen in the bottom layer was the opposite. This shows that Maluan Bay had concentrated pollution sources and poor hydrodynamic conditions, and the degradation and diffusion of nutrients were slow, thus providing sufficient nutrients for the growth of the phytoplankton in the area. The intense photosynthesis under high-temperature conditions resulted in the typical vertical stratification of dissolved oxygen, requiring special attention and control.

(5) The area ratio method can effectively reduce the effect of small-scale coastal waters with extremely high eutrophication on the overassessment of eutrophication at the broader scale, allowing the eutrophication status to be better reflected, even with limited observation data. For those bays with complex pollution mechanisms and significant regional differences in water quality damage, such as Xiamen Bay, this eutrophication assessment method has good applicability. At the same time, the methods and results presented in this study are useful to evaluate the eutrophication status in other similar bays around the world.

## Figures and Tables

**Table 1:** Coordinate and area ratio of sampling sites.

Sites	Longitude	Latitude	Frequency	Layer	Areas/km^2^	Ratio/%
XM01	118.0150	24.5458	Seasonal	Surface, bottom	6.84	1.90
XM02	118.0750	24.5525	Seasonal	Surface	16.28	4.60
XM03	118.0717	24.5233	Monthly	Surface	10.07	2.80
XM04	118.0583	24.4767	Seasonal	Surface, bottom	10.76	3.00
XM05	118.0739	24.4394	Seasonal	Surface, bottom	7.80	2.20
XM06	118.0081	24.4219	Monthly	Surface	20.15	5.70
XM07	118.0917	24.4133	Monthly	Surface	13.15	3.70
XM08	118.1481	24.4119	Monthly	Surface	16.56	4.70
XM09	118.1875	24.4408	Seasonal	Surface, bottom	17.53	4.90
XM10	118.2167	24.5000	Seasonal	Surface, bottom	31.91	9.00
XM11	118.1975	24.5439	Monthly	Surface	25.59	7.20
XM12	118.1333	24.5650	Monthly	Surface	26.93	7.60
XM13	118.1639	24.6067	Seasonal	Surface, bottom	44.38	12.50
XM14	118.2808	24.5036	Monthly	Surface	43.44	12.20
XM15	118.3492	24.5192	Seasonal	Surface, bottom	20.59	5.80
XM16	118.3850	24.5450	Monthly	Surface	31.62	8.90
XM17	118.0494	24.4483	Seasonal	Surface, bottom	11.40	3.20
Summary					355.00	100.00

**Table 2:** Grading standards and values of various assessment indicators.

Indicators			Grading Standards and Values				
			Low	Median–Low	Median	Median–High	High
			5	4	3	2	1
Pressure	COD	*P*	≤0.25	>0.25, ≤0.50	>0.50, ≤0.75	>0.75, ≤1.00	>1.00
	DIN						
	DIP						
State	Chl-a (μg/L)		≤5.00	>5.00, ≤10.00	>10.00, ≤15.00	>15.00, ≤20.00	>20.00
	Bottom DO (mg/L)		>5.00	>4.00, ≤5.00	>3.00, ≤4.00	>2.00, ≤3.00	≤2.00
	Red tide duration		<3 days, cumulatively and aperiodically	≥3 days, cumulatively and aperiodically	≥7 days, cumulatively and aperiodically or ≥3 days, continuously and periodically	≥30 days, cumulatively and aperiodically or ≥7 days, continuously and periodically	≥30 days, cumulatively or ≥14 days, continuously and periodically
Response	Changing rate of nutrients in future		−10.00%	<−10.00%	0.00	<+10.00%	+10.00%

**Table 3:** Matrix of coastal eutrophication status.

Indicators	Matrix	Status
P S R	$\left|\begin{array}{cccccc} 5 & 5 & 5 & 4 & 4 & 4\\ 5 & 5 & 5 & 5 & 5 & 5\\ 5 & 4 & 3 & 5 & 4 & 3 \end{array}\right|$	Excellent
P S R	$\left|\begin{array}{cccccccccccccccccc} 5 & 5 & 5 & 5 & 5 & 5 & 5 & 4 & 4 & 4 & 4 & 4 & 3 & 3 & 3 & 3 & 3 & 3\\ 5 & 5 & 4 & 4 & 4 & 4 & 4 & 5 & 5 & 4 & 4 & 4 & 5 & 5 & 5 & 4 & 4 & 4\\ 2 & 1 & 5 & 4 & 3 & 2 & 1 & 2 & 1 & 5 & 4 & 3 & 5 & 4 & 3 & 5 & 4 & 3 \end{array}\right|$	Good
P S R	$\left|\begin{array}{cccccccccccccccccccccccccccccc} 5 & 5 & 5 & 5 & 5 & 4 & 4 & 4 & 4 & 4 & 4 & 4 & 3 & 3 & 3 & 3 & 3 & 3 & 3 & 2 & 2 & 2 & 2 & 2 & 2 & 2 & 2 & 2 & 1 & 1\\ 3 & 3 & 3 & 3 & 3 & 4 & 4 & 3 & 3 & 3 & 3 & 3 & 5 & 5 & 4 & 4 & 3 & 3 & 3 & 4 & 4 & 4 & 4 & 4 & 3 & 3 & 3 & 2 & 3 & 3\\ 2 & 1 & 5 & 4 & 3 & 2 & 1 & 5 & 4 & 3 & 2 & 1 & 2 & 1 & 2 & 1 & 5 & 4 & 3 & 5 & 4 & 3 & 2 & 1 & 5 & 4 & 3 & 5 & 5 & 4 \end{array}\right|$	Median
P S R	$\left|\begin{array}{ccccccccccccccccccccccc} 4 & 4 & 4 & 4 & 4 & 3 & 3 & 3 & 3 & 3 & 3 & 3 & 2 & 2 & 2 & 2 & 2 & 2 & 1 & 1 & 1 & 1 & 1\\ 2 & 2 & 2 & 2 & 2 & 3 & 3 & 2 & 2 & 2 & 2 & 2 & 3 & 3 & 2 & 2 & 2 & 2 & 3 & 3 & 3 & 2 & 2\\ 5 & 4 & 3 & 2 & 1 & 2 & 1 & 5 & 4 & 3 & 2 & 1 & 2 & 1 & 4 & 3 & 2 & 1 & 3 & 2 & 1 & 5 & 4 \end{array}\right|$	Poor
P S R	$\left|\begin{array}{cccccccccccccccccc} 3 & 3 & 3 & 3 & 3 & 2 & 2 & 2 & 2 & 2 & 1 & 1 & 1 & 1 & 1 & 1 & 1 & 1\\ 1 & 1 & 1 & 1 & 1 & 1 & 1 & 1 & 1 & 1 & 2 & 2 & 2 & 1 & 1 & 1 & 1 & 1\\ 5 & 4 & 3 & 2 & 1 & 5 & 4 & 3 & 2 & 1 & 3 & 2 & 1 & 5 & 4 & 3 & 2 & 1 \end{array}\right|$	Worst

**Table 4:** Contribution weight of each sampling site for eutrophication status assessment in Xiamen seas.

	Sites	Contribution weight																	Sum	Values
Indicators		XM1	XM2	XM3	XM4	XM5	XM6	XM7	XM8	XM9	XM10	XM11	XM12	XM13	XM14	XM15	XM16	XM17		
Pressure		0.40	0.15	0.08	0.06	0.04	0.24	0.11	0.06	0.05	0.05	0.07	0.18	0.36	0.03	0.01	0.02	0.09	1.99	1.00
State	Chl-a	0.29	0.08	0.04	0.05	0.03	0.12	0.10	0.07	0.09	0.19	0.14	0.15	0.24	0.33	0.10	0.19	0.04	2.27	5.00
	Bottom DO	0.08	0.31	0.18	0.19	0.15	0.37	0.26	0.33	0.35	0.66	0.49	0.51	0.87	0.91	0.42	0.65	0.22	6.97	5.00
